# Polyphenols in the Prevention and Treatment of Colorectal Cancer: A Systematic Review of Clinical Evidence

**DOI:** 10.3390/nu16162735

**Published:** 2024-08-16

**Authors:** Laura López-Gómez, Jose Antonio Uranga

**Affiliations:** 1 Department of Basic Health Sciences, Faculty of Health Sciences, University Rey Juan Carlos (URJC), 28922 Alcorcón, Spain; laura.lopez.gomez@urjc.es; 2 High Performance Research Group in Physiopathology and Pharmacology of the Digestive System (NeuGut-URJC), University Rey Juan Carlos (URJC), 28922 Alcorcón, Spain

**Keywords:** polyphenols, flavonoids, colorectal cancer, clinical trials, curcumin, lycopene

## Abstract

Polyphenols are plant metabolites with potential anti-inflammatory and anti-proliferative effects, which may be advantageous for disorders like colorectal cancer (CRC). Despite promising in vitro and in vivo evidence, human clinical trials have yielded mixed results. The present study aimed to evaluate the clinical evidence of polyphenols for CRC prevention or treatment. A systematic review was performed according to PRISMA. Based on a PROSPERO registered protocol (CRD42024560044), online databases (PubMed and COCHRANE) were utilized for the literature search. A total of 100 studies articles were initially identified. After reviewing, 12 studies with a low risk of bias were selected, examining the effect of a variety of compounds. Curcumin demonstrated promise in various trials, mainly decreasing inflammatory cytokines, though results varied, and it did not lower intestinal adenomas or improve outcomes after chemotherapy. Neither epigallocatechin gallate nor artepillin C reduced the incidence of adenomas. Finally, fisetin seemed to improve the inflammatory status of patients under chemotherapy (5-fluorouracil). In summary, although certain polyphenols appear to exert some effect, their role in the prevention or treatment of CRC is inconclusive, and more clinical studies under more controlled conditions are needed.

## 1. Introduction

Polyphenols are a very large and diverse group of compounds with at least one aromatic ring with one or more hydroxyl groups attached. They are classified as flavonoids and non-flavonoids based on their chemical structure. Flavonoids comprise flavonols, flavones, flavanones, flavan-3-ols (catechins), anthocyanins, dihydrochalcones, chalcones and isoflavones. Non-flavonoids include phenolic acids, polyphenolic amides (capsaicinoids), stilbenes (resveratrol), lignans, and curcuminoids. All these of compounds are naturally occurring metabolites found in plants, and their concentration varies among different foods. The most important dietary sources include fruits and vegetables, cocoa, dark chocolate, soy products, cereals and wholegrains, olive oil, and drinks, such as coffee, tea, red wine, and juices [1]. There is a large body of evidence supporting polyphenols’ effectiveness in the prevention of various diseases through several biological mechanisms, including antioxidative and anti-inflammatory effects, cell cycle regulation, the induction of apoptosis, and hormonal regulation, and anti-angiogenic properties [2]. It has therefore been proposed that they may be beneficial for gastrointestinal (GI) inflammatory disorders, such as inflammatory bowel disease (IBD) [3] and colorectal cancer (CRC) [4].

CRC is one of the cancers with the highest incidence in the world, with more than 1.9 million new cases diagnosed in 2020 [5]. Less than 5% of all CRCs are considered hereditary due to underlying genetic disorders [6]. It is therefore a type of cancer that is particularly sensitive to causes, such as age, gender and ethnic background, lifestyle, an inappropriate diet, microbiota dysbiosis, lack of exercise, and chronic inflammation [7,8]. Many efforts are therefore devoted to preventing the development of CRC through health education and the promotion of good habits. However, its incidence is steadily increasing worldwide, demonstrating that new approaches to its control are urgently needed. In this regard, it is of interest to focus on controlling carcinogenesis rather than attempting to cure the disease in its terminal stage [9].

Among the many benefits attributed to polyphenols are their anti-inflammatory effects, modulating the NFκ-β and the STAT3 transcription factor pathways, and the inflammasome. They have also been associated with the stimulation of detoxification, induction of apoptosis and the inhibition of cell proliferation, reduction of oxidative stress and angiogenesis, and metastasis through the inhibition of metalloproteinases [10]. These compounds might also act as sensitizers for cancer cells or reduce the oxidative stress caused by chemotherapy [11]. Specifically, it has been shown that polyphenols like xanthohumol can inhibit the growth of human colon cancer cell lines in vitro even better than that of some chemotherapeutic drugs like 5-fluorouracil. Other compounds have shown a synergistic effect in combination with chemotherapy, although differences in responses were observed depending on the tumor line tested [12]. Furthermore, these compounds reach the intestine where they or their metabolites may act as substrates or cofactors in biochemical and enzymatic reactions, inhibiting enzymes, ligands for cell receptors, and scavengers and promoting beneficial gastrointestinal bacteria, serving as fermentation substrates, and selectively inhibiting harmful intestinal bacteria [10].

Despite experimental evidence, in vitro and in vivo, and the numerous epidemiological studies indicating that consuming diets high in flavonoids is linked to a lower risk of developing cancer [10], the association between polyphenol intake and colorectal cancer risk have not yielded consistent results [4]. In this way, several case studies have suggested an inverse association between the high intake of certain compounds, mainly flavonoids, and CRC. For example, a significant effect of flavonols, catechins, and flavones on CRC has been described regardless of the cancer site [13,14,15]. However, other authors have observed differences depending on whether the primary tumor is in the colon, where high doses of flavonols, such as quercetin, would be beneficial, or in the rectum, where apigenin, a flavone, would be beneficial [16]. On the other hand, there are several prospective studies that have found no relationship between flavonoid intake and the occurrence of CRC [4], regardless of sex [17] or smoking status [18], although catechin intake could play a role in obese male patients [19].

To clarify the current situation on this subject, we aim to provide, in this paper, a systematic review of the clinical trials performed with polyphenols for the prevention and treatment of CRC.

## 2. Materials and Methods

This article is a systematic review and was performed in accordance with the guidelines provided by the PRISMA statements [20,21,22]. The protocol was registered in the international database PROSPERO for systematic reviews (CRD42024560044) to prevent the possibility of duplication and decrease the information bias by allowing for a comparison of the completed review with the planned protocol. This review tries to answer the following research question: is there evidence that the use of polyphenols and flavonoids in the diet or any of its formulations could be useful in preventing the development or progression of CRC.

### 2.1. Search Strategy and Selection Criteria

The search for information was conducted between 1 May 2023 and 1 May 2024. For the search strategy, the open-access search tool PubMed and COCHRANE database were used to identify eligible studies. As selection criteria, in this study, we have focused only on human clinical trials in which flavonoids have been used as a preventive method or as an adjunct to the treatment of colorectal cancer. The search for all relevant articles was performed by evaluating the title, abstract, and full text. We also conducted a search using a type of non-probability sampling called “snowballing” to identify additional studies through the bibliographic references of specific publications [23].

To facilitate the search query, general terms referring to polyphenols, flavonoids, and the different subfamilies into which they are divided (flavonoids, polyphenols, flavonols, flavanones, isoflavones, flavanols, flavones, or anthocyanins) were used. In addition, terms referring to specific widely used compounds were included, because of their interest: quercetin, myricetin, kaempferol, curcumin, resveratrol, procyanidins, epicatechin, catechin, chlorogenic acid, caffeic acid, cinnamon, or gingerol. In the query utilized for returning data from the databases, these terms were cross-referenced with colorectal cancer OR colitis using the AND Boolean operator and limiting the search to clinical trials in the advance options.

To ensure all data were appropriately extracted, the authors reviewed and assessed the results independently. The resulting articles were initially tested for duplicates across the databases, to remove them from the pool. Then, an initial reading of the title and the abstracts was made to detect and remove irrelevant articles included during the initial search. Finally, the complete contents of the remaining articles were read, and the reference lists of the articles were reviewed to detect relevant current articles that were not included in the search procedure. Inclusion and exclusion criteria were applied to narrow down the result set and to include only relevant studies in the revision.

### 2.2. Inclusion and Exclusion Criteria

As inclusion criteria, no year of publication restrictions was applied; the relevant studies for reviewing had to be written in English, referring to the effects of polyphenol or flavonoid administration on colorectal cancer prevention or progression, and should only refer to human populations regardless of age, gender, or ethnicity. Both, pure isolated flavonoid intervention and controlled dietary interventions were considered. Healthy patients who developed cancer during the period of flavonoid administration, patients at all stages of colorectal cancer development, with presence of adenomatous polyps, or those with a family history of the disease were included.

As exclusion criteria, publications in other languages were removed. Case reports, editorial opinions, congress abstracts, conference proceedings, and trial registry records or trial protocols were eliminated, as they were considered not to contain complete information about the studies. Cell culture studies were not included due to their difficulty to compare the results with human population studies. Animal studies were also considered irrelevant and omitted. Articles that do not specifically address therapeutic aspects of polyphenols or flavonoids in CRC and only focus on determining safe and appropriate doses to administer in trials were discarded. Failure to meet at least one inclusion criterion classified a study as not interesting for inclusion.

### 2.3. Data Extraction

Data extraction was performed independently by two researchers using a piloted form onto a Microsoft Excel 365 spreadsheet. The following details were extracted from each study: title of the article, authors, year of publication, country, gender of patients, number of patients involved in the study, range of age, health status or cancer stage, type of study, type of flavonoid tested (route of administration, dose/concentration used, and duration of treatment), and objectives and main conclusions of the study. If the relevant data were not provided, we attempted to contact the authors to obtain the information. If, despite everything, it was not possible to obtain the necessary information, the study was eliminated from the review.

### 2.4. Assessment of Risk Bias

To help to establish the transparency of results, both investigators, using the Cochrane Risk of Bias Tool and independently, assessed risk of bias at the individual study level [21]. The disagreement among the evaluators was resolved by consensus in a final discussion. Risk of bias was categorized as low, unclear, or high based on six domains including the following: random sequence generation (selection bias), allocation concealment (selection bias), blinding of participants and personnel (performance bias), blinding of outcome assessment (detection bias), incomplete outcome data (attrition bias), selective reporting (reporting bias), and other sources of bias. Based on these six domains, each trial was assigned an overall risk of bias: low risk (low for all key domains), high risk (high for one or more key domains), or unclear risk (unclear for one or more key domains). “Unclear risk of bias” denotes insufficient information to determine the possibility of bias. Studies with one domain with a high risk of bias or four domains with an unclear risk of bias were considered as a high risk of bias [21].

## 3. Results

### 3.1. Article Selection

**Figure 1:**
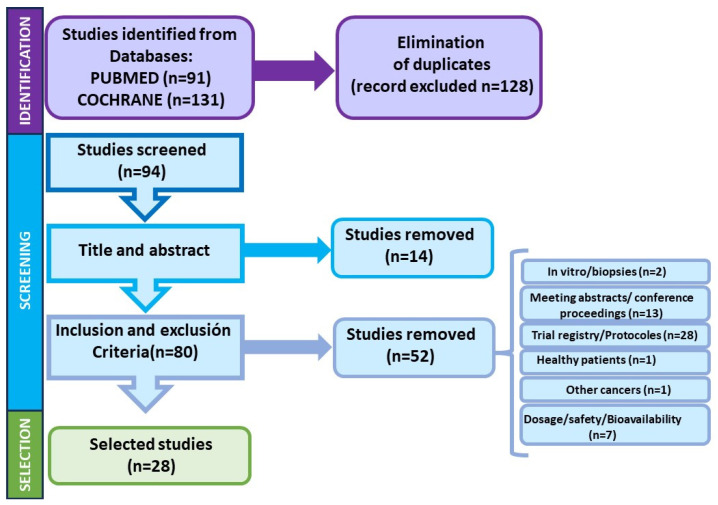
Schematic representation of the selection process of scientific articles.

Figure 1 summarizes the process of identification of the studies for this systematic review.

In the first literature search, 91 articles were found in PubMed and 131 in COCHRANE that were likely to be relevant to the review, and the first step of eliminating duplicates excluded 128 articles, leaving only 94 for the next phases of the screening.

In the next step, the title and abstract of the pre-selected articles were reviewed, and 14 articles were eliminated for the following reasons: no reference to colorectal cancer (n = 5), in vitro cell culture studies (n = 2), treatment of other unrelated pathologies (n = 2), or studies performed in healthy population (n = 5). After this step, 80 items were left to continue with the scrutiny.

Finally, the complete text of the articles was read to ensure that they contained information relevant to the study, and the inclusion and exclusion criteria were applied. The application of the exclusion and inclusion criteria led to the elimination of another 52 articles. The specific reasons were meeting abstracts or short communications (n = 10), conference proceedings (n = 3), trial registry records (n = 27), protocol description (n = 1), studies in healthy patients not directly related with colorectal cancer (n = 1), in vitro studies or using biopsies (n = 2), or other types of cancers (n = 1). Lastly, articles that do not specifically address the therapeutic or preventive aspects of polyphenols and flavonoids in CRC (dosage determination, safety, bioavailability) (n = 7) were also removed.

After applying all the steps, 28 articles were finally selected for systematic review and were subjected to a bias screening (see Table 1).

### 3.2. Data Extraction

#### 3.2.1. Characteristics of the Selected Studies

Among the 28 selected studies, 12 of them aimed to evaluate whether the compounds were able to prevent the development of CRC in patients with risk factors, such as a family history of CRC or premetastatic lesions, while 16 focused on their own potential to treat CRC. On the other hand, 26 studies used the pure compounds or preparations with known concentrations of polyphenols, while 6 of them performed dietary interventions in which the compounds were included as part of the usual intake of the patients.

Studies were conducted in 9 different countries: USA (12), UK (5), Netherlands (2), Iran (2), Italy (2), Taiwan (2), China (1), Japan (1), and Germany (1).

#### 3.2.2. Patients

Although the studies have used patients of all ages, from 18 to 85 years, the average age was 51. In general, the studies included patients of both sexes, except for some cases in which only men [35] or only women [37,44] were used. In one case, the sex of the patients was not specified [25]. In the studies in which pure compounds were used, the total number of patients evaluated was 1408, whereas in the dietary interventions involving long-term follow-up, the number was higher, 161,946 patients.

### 3.3. Risk of Bias

The result of the analysis of bias is graphically represented in Figure S1 (see Supplementary Materials).

Random sequence generation was identified in 16 trials and classified as having a low risk of bias in this domain [26,30,31,32,33,34,35,36,38,39,40,41,42,43,44,46,50].

Sixteen studies reported data on allocation concealment [26,30,31,32,33,34,35,36,38,39,40,41,42,43,44,50]. The blinding of participants and personnel was described in 15 studies [26,30,31,32,33,34,35,36,38,40,41,42,43,44,47,50], but the rest of studies did not apply any blinding method, or this was unclear. Outcome assessment blinding was thoroughly described in 14 studies [26,30,31,32,33,34,35,36,38,40,41,42,43,44,50].

Twenty-three studies were considered as having a low risk of bias in terms of the incomplete outcome [4,24,25,26,29,30,31,32,33,35,36,38,39,40,41,42,43,44,45,46,47,48,49,50]. No selective reporting bias was detected in any of the studies, although in some cases, its presence is unclear [25,27,28,29,32,34,37]. Other sources of bias related to participant dropout data or the imbalance between the evaluated blocks were also considered in the analysis.

In summary, 14 studies were classified as having a high risk of bias due to having at least one high-risk domain among the six key domains, while 2 studies had an unclear risk of bias. Finally, only the 12 studies considered as having a low risk of bias were selected for the discussion of this review [26,30,31,33,35,36,40,41,42,43,44,50].

## 4. Discussion

After evaluating the articles found in the search, only 12 of them have been selected in this systematic review to explore the possibilities of the clinical use of polyphenols for the treatment or prevention of CRC.

Artepillin C, a prenylated derivative of p-coumaric acid, is one of the major phenolic compounds found in Brazilian Green Propolis, an important honeybee product. Different vegetal materials are collected by honeybees and mixed with wax and other substances to obtain this final product, called propolis. *Baccharis dracunculifolia* is the major plant source with several pharmacological benefits, including anti-cancer effects [51]. Preclinical studies have shown that this compound has good potential for the treatment of CRC. Artepillin C, added to human colon cancer cells, dose-dependently inhibited cell growth, inducing G (0)/G (1) cell cycle arrest [52]. In animal models, dietary artepillin C was shown to suppress the formation of aberrant crypt foci induced by azoxymethane in the colon, through the activation of the antioxidant-responsive element and induction of phase II enzymes in liver [53]. Similarly, in a Wistar rat model using 1,2-dimethylhydrazine induction, artepillin C decreased aberrant crypt foci amounts and prevented crypt cell clonal expansion [54]. However, a pilot study on a Brazilian propolis extract containing artepillin C found no evidence of effectiveness in preventing early-stage colon cancer changes and raised concerns about potential muscle tissue side effects, including the myocardial tissue [26]. The small sample size (only 15 patients treated) could be a reason for these results. In addition, poor bioavailability limits the biological effects of phenolic compounds, including artepillin C. In a study performed on Wistar rats, artepillin C presented low absorption and bioavailability due to greater susceptibility to hepatic elimination [55].

Curcumin, the active ingredient of the *Curcuma longa* plant, has triggered the interest of researchers as an antioxidant, anti-inflammatory, and anticancer agent. Mainly, curcumin enhanced modulated tumor cell apoptosis by modulating levels of B-cell lymphoma protein 2-associated X (Bax) and B-cell lymphoma protein 2 (Bcl-2), two important molecules in the apoptotic pathway, and by activating p53 [56]. Another major pathway that curcumin targets is the matrix metalloproteinase gene expression [57]. Notably, curcumin’s pro-apoptotic effects are specific to cancer cells, with no significant impact on normal cells [58]. Several clinical trials have been performed using curcumin alone or combined with other products with very promising results. Its potential reaches from acting itself as an anticancer compound [30] to exerting preventive action in patients with adenomas [31,33] or as a coadjuvant to conventional chemotherapy [35,36].

Regarding its anticarcinogenic activity, clinical trials has shown that curcumin administration upregulates p53 expression in tumor tissue. P53 is a tumor suppressor protein responsible for a wide range of signaling events within cells, leading to necrosis or apoptosis. In this way, curcumin induces apoptosis in CRC cells [30]. In addition, its administration improves the general condition of the patients with CRC by increasing body weight and decreasing levels of serum TNF-α. Interestingly, TNF-α is elevated in extracellular vesicles from CRC patient serum samples and is related to the development of aggressive forms of CRC. Currently, it is known that the extracellular vesicles are critical for CRC progression by enabling intercellular communication and controlling the microenvironment of tumor cells. Functional experiments revealed that extracellular vesicles containing TNF-α promote CRC cell metastasis via the nuclear factor kappa B (NF-κB) pathway [59]. The positive effects of curcumin on TNF-α levels may also contribute to its anticancer action.

The role of curcumin as a preventive agent in the development of CRC has also been studied. However, in a preventive trial, pure curcumin (3000 mg daily/12 months) did not produce any in mean number or size of lower intestinal tract adenomas. An insufficient dose of curcumin in this trial might be, among others, a major factor for these findings [31]. However, the administration of curcumin (200 mg daily/6 weeks) combined with anthocyanin (500 mg) to patients with pre-metastatic adenomas produced a reduction in the expression of NF-κB in their biopsies [33]. NF-κB has a key role in cancer-related processes, such as cell proliferation, apoptosis, angiogenesis, and metastasis [60]. Animal studies suggest that NF-κB is implicated in the early stages of the formation of adenomas with malignant potential formation [61]. But the fact that in this trial two compounds are combined makes it difficult to determine which one is responsible for the positive effects. In addition, there are major methodological differences between these two studies concerning doses and durations. The first study used 3000 mg of curcumin daily for 12 months [31], while in the second one, both the dose and duration were lower, 200 mg of curcumin daily for 6 weeks [33].

Curcumin has also been of interest from the point of view of an adjuvant, combined with established anticancer therapies. However, the addition of curcumin (8 g daily/6 weeks) to patients receiving preoperative chemoradiation therapy did not improve outcomes. In addition, the serum curcumin concentration was very variable, suggesting changes in the bioavailability of curcumin [36]. In this way, the administration of curcuminoids (500 mg day/8 weeks), including Biopiperine, a bioavailability enhancer, had a positive impact on levels of pro- and anti-inflammatory cytokines and quality of life in patients with CRC undergoing chemotherapy. Curcumin improved the erythrocyte sedimentation rate, interleukin (IL)-1-a, and serum levels of C-reactive protein in CRC subjects and increased quality of life and functional scales compared to a placebo [35]. The inclusion of this compound to increase the bioavailability of curcumin may have contributed to the positive results in this case.

Curcumin has therapeutic potential for colorectal CRC through effects on multiple molecular targets, but its low bioavailability limits its clinical translation and is an important factor to be considered in the studies. Its low solubility, short half-life, and low bioavailability, with poor absorption, rapid metabolism, and rapid systemic elimination, reduces its applicability [62,63].

Isoflavones are phytoestrogens, naturally occurring nonsteroidal phenolic plant compounds, chemically comparable to vertebrate steroid estrogens. They can be obtained from different sources as soy or red clover [64]. Soy isoflavones (83 mg/12 months) used as a preventive did not reduce colorectal epithelial cell proliferation in a dietary intervention performed in a study population with previous adenomatous polyps [41]. There are studies in Asian populations that support the relationship between the consumption of large amounts of these compounds and a lower incidence of CRC [65,66,67]. However, the intervention by Adams et al. (2005) [41] was much shorter and performed in U.S. populations whose diet is much lower in isoflavones than Asians. Red clover is another source of isoflavones, mainly biochanin A and formononetin, which are metabolized to genistein and daidzein [40]. Interestingly, since isoflavones have weak estrogenic activity, they could produce an estrogen-like decrease in serum insulin-like growth factor (IGF)-I concentrations [40]. The elements of the IGF system have an important function in the regulation of the growth, proliferation, and survival of cells. Prospective epidemiological studies indicate that higher circulating concentrations of IGF-I and II and reduced concentrations of insulin-like growth factor-I-binding proteins IGFBP-1 and IGFBP-2 are associated with an increased CRC risk [68]. Although at the doses used (84 mg daily/8 weeks), isoflavone supplementation did not influence circulating IGF concentrations in men at a high risk of CRC, a relative decrease in serum total IGF-I concentrations was found in equol producers only. An individual’s ability to produce equol, a potent estrogenic compound formed during the metabolism of daidzein, seems to be a relevant factor in the effect of isoflavones on IGF system components. As this study was carried out only in a male population, the researchers extended it to women with an increased risk of CRC and showed consistently that isoflavone administration did not significantly affect circulating levels of IGF system components or influence serum concentrations and tissue mRNA expression [42]. Therefore, the evidence for an association between isoflavones from red clover and reduced colorectal cancer risk is inconclusive.

Epigallocatechin gallate (EGCG) is a polyhydroxy phenolic compound extracted from tea, for which antitumor and radio-sensitizing effects on cancer cells has received attention [69,70,71]. This compound has been shown to inhibit in vitro cell proliferation and induce apoptosis in CRC cells in part by modulating the activities of various receptor tyrosine kinases. Additionally, EGCG activates stress signals, such as JNK and p38 mitogen-activated protein kinase (MAPK) and induces apoptosis in CRC cell lines [71]. The mechanisms underlying the anti-tumor efforts of EGCG may include the inhibition of epidermal growth factor and IGF/IGF1R signaling pathways as shown in animal and human studies [72,73]. In view of the promising results obtained in preclinical studies, a preventive assay tested the efficacy and safety of Polyphenon E (Poly E), a polyphenol preparation with EGCG (780 mg daily/6 months), in patients with rectal aberrant crypt foci (ACF), precursors of CRC [43]. Poly E was well tolerated and did not present significant toxicity at the dose studied but did not reduce total number of ACFs. The study presented limitations due to a small sample size of only 19 patients and a study population restricted to high-risk CRC subjects. These limitations could impact the generalizability of the findings [43].

Fisetin (3,3′,4′,7-tetrahydroxyflavone) is a dietary flavanol present in fruits and vegetables, such as grape, apple, persimmon, strawberry, cucumber, or onion, and has anti-inflammatory, apoptotic, anti-oxidative properties. It has shown good results in several preclinical studies as an adjuvant in chemotherapy or radiotherapy. In vitro models have shown that fisetin induces apoptosis by suppressing autophagy and down-regulating nuclear factor erythroid 2-related factor 2 (Nrf2) [74] and may represent a relevant strategy in cancer pathogenesis and resistance cases. Fisetin improves radiosensitivity by blocking the repair of radiation-induced DNA double-strand breaks [75]. It is also capable of enhancing the radiosensitivity of p53-mutant human CRC cells [76]. A combination of fisetin with antitumor drugs, such as 5FU or oxaliplatin and irinotecan, increases the efficacy of these drugs and could be useful in cases of treatment resistance [77,78]. This compound could also be used for inflammation-related tumor progression. The administration of fisetin to patients with CRC (Stages I–II) revealed a decrease in IL-8 and C-reactive protein concentrations, improving the inflammatory status in CRC patients. In this way, the potential of this compound to increase the maximum-tolerated dose of chemotherapeutic agents may increase the chance of survival and the quality of life and associated comorbidities [44].

Most of the studies selected in this review opt for the use of pure compounds, as this allows for a better dose control. However, there have been studies with less controlled formulations as the herbal agent MB-6, as a potential adjunct to 5-FU–based chemotherapy [50]. MB-6 is a botanical product manufactured by Microbio Co., Ltd., in Taipei, Taiwan. It is formulated from diverse plants: fermented soybean extract (MicrSoy-20), green tea extract (*Camellia sinensis* O), *Antrodia camphorata mycelia*, spirulina (*Arthrospira platensis),* grape seed extract (*Vitis vinifera*), and curcumin extract (*Curcuma longa* L). MB-6 treatment produced a lower disease progression rate in CRC patients and reduced the incidence of adverse events and occurrence of increased serum creatinine [50]. However, the fact that MB-6 is a combination of several different herbs makes it extremely difficult to determine which of the compounds administered is responsible for the benefits it provides, as well as the proper dosage.

## 5. Conclusions

Some polyphenols have shown anticarcinogenic effects, and their benefits have been demonstrated in many in vitro and epidemiological studies, where they appear to exert anti-oxidative, anti-inflammatory effects, and they have also been associated with the stimulation of apoptosis and the inhibition of cell proliferation, among other interesting properties. However, the limited number of clinical trials conducted with each compound does not allow us to be certain that they play a significant role in the prevention or treatment of CRC. There is still a need for more clinical trials before they can be recommended as effective elements for the prevention or clinical treatment of colorectal cancer.

This systematic review underscores the complexity of translating preclinical success to human treatments and the need for more controlled studies to determine effective doses and formulations of polyphenols.

## Figures and Tables

**Table 1:** Selected articles for the systematic review (28). The 12 articles that finally passed the bias evaluation and were used for the discussion are underlined for identification purposes.

Polyphenol	Administration	Country	Patients	Gender	Age	Type of Study	Objective	Findings	Reference
Flavopiridol (synthetic flavone)	Isolated compound 50 mg/m^2^/day via a 72 h continuous infusion every 14 days.	USA	20 patients Advanced CRC unresectable adenocarcinoma of the colon or rectum	Both	42–80	Phase II trial of flavopiridol in previously untreated patients with advanced CRC	Examine flavopiridol as a chemotherapy modulating agent	No anticancer activity by itself	[24]
Flavopiridol (synthetic flavone)	Isolated compound (40–90 mg/m) 2–3 h after irinotecan and before 5FU	USA	31 with CRC, 43 other tumors. No control group Plasma tumor biopsies	Both	19–83	Open-label, non-randomized dose escalation study to Clinical trial Phase I	Determine if FOLFIRI in combination with flavopiridol presents therapeutic activity	Flavopiridol enhance the effects of FOLFIRI	[25]
Artepillin	165 μ mol artepillin C (extract of Propolis)/day 3 months	Japan	30 patients (15 treatment, 15 placebo) Premetastatic adenomas Biopsies	Both	40–75	Randomized, placebo controlled, double-blind trial	Evaluate the effects of artepillin C on colon cancer formation	Not effectiveness in preventing colon cancer. Detrimental side effects on muscle cells	[26]
Curcuma extract	Capsules at doses between 440 and 2200 mg/day, containing 36–180 mg of curcumin; 4 months.	UK	15 patients Advanced CRC refractory to chemotherapy Colorectal tissue biopsy	Both	42–72	Dose-escalation pilot study	Investigate potential biomarkers of the systemic efficacy of curcumin: GST and M1G	440 mg of Curcuma extract produced a decrease in lymphocytic GST activity	[27]
Curcumin	450, 1800, or 3600 mg of curcumin daily, for 7 days before surgery	UK	12 patients (7 male, 5 female) No placebo CRC Colorectal tissue biopsy	Both	47–72	Dose-scalation study	Test pharmacologically active levels of curcumin measured based on effects on levels of M1G and COX-2	3600 mg of curcumin reduced M1G levels. COX-2 protein levels were not affected	[28]
Curcumin + Quercetin	Curcumin 480 mg and quercetin 20 mg orally 3 times a day 3–6 months	USA	5 patients (3 men, 2 women) Adenomatous polyposis	Both	22–54	Proof-of principle	Evaluate the effect of curcumin and quercetin in familiar adenomatous polyposis	Decrease in polyp number and size after a mean of 6 months of treatment	[29]
Curcumin	Curcumin capsules 360 mg curcumin, thrice/day during the period ahead of surgery	China	126 patients Curcumin (37 female and 26 male) Vehicle (34 female 28 male) CRC Biopsy/blood	Both	34–82	Randomized controlled trial	Effects of treatment with curcumin on CRC patients	Increased body weight. Decreased serum TNF-α levels. Increased apoptosis of tumor cells and enhanced expression of p53	[30]
Curcumin	Pure curcumin (1500 mg orally, twice/day), or placebo, 12 months.	USA	44 patients (21 treatment, 23 placebo), Familiar adenomatous polyposis	Both	18–35	Double-blind, randomized trial	Determine safety and efficacy of curcumin	Few adverse effects No difference in mean number or size of adenomas	[31]
Curcumin	2 g oral curcumin daily during chemotherapy	UK	27 patients (9 FOLFOX, 18 CUFOX (curcumin + FOLFOX)) CRC patient plasma	Not specified	53–78	Phase IIa open-labeled randomized controlled trial	Assess safety, efficacy, and CXCL1 in patients receiving CUFOX	Curcumin did not significantly alter CXCL1 over time. Curcumin is a safe and tolerable adjunct to FOLFOX chemotherapy	[32]
Curcumin + Anthocyanin	Curcumin: 100 mg, 2 times/day Anthocyanin: 500 mg, 2 times/day	Italy	29 patients (treated: 10 men + 5 women); 14 placebo: 7 + 7) Pre-metastatic adenoma biopsy	Both	Treatment 70.8 placebo 67.9	Proof-of principle	Evaluate the preventive effect of curcumin combined with anthocyanin	Reduction in NF-κB expression in adenoma tissue. Potentially favorable	[33]
Curcumin + Anthocyanin	Daily dose of 1 g of curcumin and 1 g of anthocyanin	Italy	29 patients (15 treatment, 14 placebo), Adenomatous polyp	Both	18–85	Randomized, double-blind, placebo-controlled, phase II presurgical trial	Analyze the effects on circulating biomarkers of inflammation and metabolism	No modulation of circulating biomarkers. Increase in IL-6	[34]
Curcuminoids	Curcuminoid capsules (500 mg/day), for 8 weeks.	Iran	64, treated 59, placebo CRC (stage 3) Blood samples	Both	58–63	Double-blind placebo-controlled trial	Explore the effects of curcuminoids on pro- and anti-inflammatory cytokines and quality of life in patients undergoing chemotherapy	No improvement in cytokines. Decrease in erythrocyte sedimentation rate and C-reactive protein. Improved quality of life	[35]
Curcumin	Isolated compound 4 g orally, twice daily during radiotherapy and 6 days after	USA	22 patients (13 men, 8 treatment + 5 placebo and 9 women, 7 treatment + 2 placebo). Rectal cancer Biopsy/plasma	Both	28–75	Phase II randomized double blinded trial	Evaluate the efficacy of curcumin with pre-operative chemoradiation for rectal cancer	No increase in response rates in chemoradiation therapy due to unpredictable bioavailability of curcumin	[36]
Resveratrol	8 daily doses of resveratrol at 0.5 or 1.0 g prior to surgical resection	UK	20 patients (9 male–11 female) CRC Blood/colorectal biopsy	Both	46–83	Clinical trial	Analyze the affect of resveratrol on Ki67 expression	Reduction in tumor cell proliferation. Decreased tumor cell Ki-67 staining	[37]
Resveratrol	SRT501, micronized resveratrol 5.0 g daily for 14 days	UK	9 Patients (6 treatment, 3 placebo) Stage IV CRC and hepatic Metastases Plasma/hepatic tissue	Both	>18	Phase I, randomized double-blind clinical trial	Asses the safety, pharmacokinetics, and pharmacodynamics of the formulation	Cleaved caspase-3, a marker of apoptosis, was increased in malignant hepatic tissue	[38]
Silymarin (4 compounds: silybin, isosilybin, silychristin, silydianin)	Silymarin capsules (150 mg) 3 times/day for 7 days from the beginning of chemotherapy	Taiwan	70 patients (35 + 35) Metastatic CRC FOLFIRI plus bevacizumab	Both	20–80	Prospective open label pilot study	Evaluate the effect of silymarin supplementation reducing the toxicity of chemotherapy	Fewer adverse effects like diarrhea and nausea. No significant differences in hepatic toxicities	[39]
Isoflavones	8-week isolated (84 mg/d). isoflavone tablets	Netherlands	37 patients, 17 intervention 20 placebo Family history of CRC or a personal history of colorectal adenomas	Men	40–75	Randomized, placebo-controlled, double-blind	Investigate the effect of an isolated isoflavone supplementation serum concentrations of total IGF-I	No influence on circulating IGF concentrations in men at a high risk of CRC. IGF-I-lowering effect in equol producers only	[40]
Soy Isoflavones	Soy isoflavones Treatment (83 mg isoflavones Control group (3 mg isoflavones).	USA	125 Patients Adenomatous colorectal polyps Biopsies colon and rectum	Both	50–80	12-month randomized, double-blinded, placebo-controlled dietary intervention	Test if soy isoflavones decrease epithelial cell proliferación	Soy isoflavones do not reduce colorectal epithelial cell proliferation	[41]
Isoflavone	8-week supplementation with red clover-derived isoflavones (84 mg/d)	Netherlands	34 Patients 15 isoflavone 19 placebo Colorectal adenomas or family history of CRC Plasma/biopsy	Women	50–75	Randomized, placebo-controlled, double-blinded, crossover trial	Investigate the effect of isolated isoflavones on IGF system components	No changes in circulating levels of IGF system components or in colorectal tissue mRNA expression	[42]
Epigallocate chin gallate	Polyphenon E (Poly E, epigallocatechin gallate); 6 months of ora (780 mg EGCG) daily	USA	39 patients 19 Poly E 20 placebo Rectal ACF (putative precursors of CRC)	Both	>40	Randomized, double-blinded, and placebo-controlled trial	Study preventive efficacy and safety of Poly E in subjects with rectal ACF	No reduction of rectal ACF number relative to placebo	[43]
Fisetin	100 mg capsule of Wax-Tree-derived fisetin/day 7 consecutive weeks	Iran	37 patients 18 treated, 19 control CRC Stages II-II Blood samples Chemotherapy	Both	55	Double-blind, randomized placebo-controlled clinical trial	Assess the efficacy of fisetin supplementation the inflammatory status and MMP levels	Fisetin reduced levels of IL-8 and MMP-7. Act as complementary antitumor agent	[44]
Flavonoid mixture	Daily dose of 20 mg apigenin and 20 mg epigallocathechin-gallate	Germany	87 patients, 31 treated 56 control group Resected colon cancer and polypectomy	Both	69–82	Controlled clinical trial. Prospective and observational cohort study	Investigate biological prevention with flavonoids the recurrence risk of neoplasia	Sustained long-term treatment could reduce the recurrence rate of colon neoplasia	[45]
Diet	Intake of the six flavonoid subgroups 4 years	USA	1905 Patients (control 947; intervention 958) Colorrectal adenomas	Both	61	Randomized dietary intervention	Examine the effectiveness of a low-fat, high-fiber, high-fruit, and high-vegetable diet on adenoma recurrence	High intake of flavonols was associated with a decreased risk of advanced adenoma recurrence	[46]
Diet	Intake of flavonols (quercetin, kaempferol, and myricetin) and flavones (apigenin and luteolin).	USA	38,408 women, 3234 developed cancer after 11 years of follow-up All kinds of cancers/305 CRC	Women	>45	Prospective study	Investigate the association between the intake of selected flavonoids and risk of cancers	There was no association between intake of flavonoid-rich foods and the incidence of cancers. No prevention	[47]
Diet	Low-fat, high-fiber, high-fruit and vegetable, diet Flavonols isorhamnetin, kaempferol, and quercetin	USA	872 participants Histologically confirmed colorectal adenomas Blood	Both	>35	Polyp Prevention Trial 4-year	Examine the effectiveness of a low-fat, high-fiber, high-fruit, and vegetable diet on adenoma recurrence	High flavonol intakes decrease IL-6 concentrations. Reduction of the risk of adenoma recurrence	[48]
Diet	Intake of lignan and proanthocyanidin	USA	1859 patients control 930; intervention 929 Presence of adenomas	Both	55	Randomized, nutritional intervention trial	Evaluate if consumption of a high-fiber, high-fruit, high-vegetable, and low-fat diet would decrease the risk of adenoma recurrence	High lignan intake may increase the risk of adenoma recurrence in women	[49]
Diet	MB-6 6 capsules of 320 mg each, 3 times/day	Taiwan	60 patients 29 treated 31 placebo CRC Follow-up assessment 77 weeks	Both	63	Proof-of-concept clinical study 77 weeks of follow-up	Test if MB-6 would increase the effectiveness of chemotherapy (FOLFOX)	MB-6 group had a lower disease progression rate and presented less adverse events and a decreased serum creatinine	[50]
Diet	Flavonoid subclasses (flavonols, flavones, flavanones, flavan-3-ols, and anthocyanins) 26 years	USA	42,478 male and 76,364 female 2519 CRC cases (1061 men, 1458 women) Evaluation of cancer risk	Both	30–75	Polyp Prevention Trial	Examine if higher dietary intakes of flavonoids was associated with a lower risk of CRC	Higher dietary intakes of flavonoids was not associated with a lower risk of CRC.	[4]

Abbreviations: 5FU, 5-fluorouracil; ACF, aberrant crypt foci; COX-2, cyclooxygenase-2; CRC, colorectal cancer; CXCL1, chemokine (C-X-C motif) ligand 1; EGCG, epigallocatechin gallate; GST, glutathione S-transferase; IL, interleukin; M1G, pyrimido [1,2-a] purin-10 (3H)-one; MB-6, extracts from fermented soybean, green tea, *Antrodia camphorata mycelia*, spirulina, grape seed, and curcumin; MMP, matrix metalloproteinase; mRNA, messenger ribonucleic acid; NF-κB, nuclear factor kappa B; Poly E, Polyphenon E; TNF, tumor necrosis factor; UK, United Kingdom; USA, United States of America.

## Data Availability

The data presented in this study are available on request from the corresponding author and will also be available at the corresponding authors’ institutional repository (URJC).

## Supplementary Materials

The following supporting information can be downloaded at: https://www.mdpi.com/article/10.3390/nu16162735/s1, Figure S1: Graphical representation of the bias analysis according to seven key domains.
