# Supplementary material for: Polyphenols in the Prevention and Treatment of Colorectal Cancer: A Systematic Review of Clinical Evidence
Source: Nutrients. 2024 Aug 16;16(16):2735. doi: 10.3390/nu16162735 (PMC11357634; doi:10.3390/nu16162735)
Supplement: Supplementary file 1 [file nutrients-16-02735-s001.zip › nutrients-3120536-supplementary.pdf]

nutrients

Systematic Review

Polyphenols in the Prevention and Treatment of Colorectal Cancer: A Systematic Review of Clinical Evidence

Laura López-Gómez [1,2] and Jose Antonio Uranga [1,2,*]

[1] Department of Basic Health Sciences, Faculty of Health Sciences, University Rey Juan Carlos (URJC), 28922 Alcorcón, Spain; laura.lopez.gomez@urjc.es

[2] High Performance Research Group in Physiopathology and Pharmacology of the Digestive System (NeuGut-URJC), University Rey Juan Carlos (URJC), 28922 Alcorcón, Spain, 28922 Alcorcón; Spain

* Correspondence: jose.uranga@urjc.es; Tel.: +34-91-488-8621

Keywords: polyphenols; flavonoids; colorectal cancer; clinical trials; curcumin; lycopene

References	Random sequence generation	Allocation concealment	Blinding of participants and personnel	Blinding of outcome Assessment	Incomplete outcome data	Selective reporting	Other sources of bias	Overall quality
[24]	-	-	-	-	+	+	?	Hight
[25]	-	-	-	-	+	?	-	Hight
[26]	+	+	+	+	+	+	+	Low
[27]	-	-	-	-	-	?	?	Hight
[28]	-	-	-	-	?	?	?	Hight
[29]	-	-	-	-	+	?	?	Hight
[30]	+	+	+	+	+	+	+	Low
[31]	+	+	+	+	+	+	+	Low
[32]	-	+	-	-	?	?	?	Hight
[33]	+	+	+	+	+	+	+	Low
[34]	+	+	+	+	?	?	?	Unclear
[35]	+	+	+	+	+	+	+	Low

[36]	+	+	+	+	+	+	+	Low
[37]	-	-	-	-	?	?	+	Hight
[38]	+	+	+	+	+	+	?	Unclear
[39]	+	+	-	-	+	+	+	Hight
[40]	+	+	+	+	+	+	+	Low
[41]	+	+	+	+	+	+	+	Low
[42]	+	+	+	+	+	+	+	Low
[43]	+	+	+	+	+	+	+	Low
[44]	+	+	+	+	+	+	+	Low
[45]	-	?	-	?	+	+	?	Hight
[46]	+	-	-	-	+	+	+	Hight
[47]	-	-	+	?	+	+	+	Hight
[48]	-	-	?	?	+	+	+	Hight
[49]	-	-	?	?	+	+	+	Hight
[50]	+	+	+	+	+	+	+	Low
[4]	-	?	-	-	+	+	+	Hight

Figure S1: Graphical representation of the bias analysis according to seven key domains. Hight risk of bias, Low risk of bias, Unclear risk of bias
